# A Whole W-Band Multi-Polarization Horn Antenna Based on Boifot-Type OMT

**DOI:** 10.3390/mi15030385

**Published:** 2024-03-13

**Authors:** Yun Zhao, Bo Zhu, Jiangqiao Ding, Sheng Li

**Affiliations:** 1 Jiangsu Collaborative Innovation Center of Atmospheric Environment and Equipment Technology, School of Electronic & Information Engineering, Nanjing University of Information Science and Technology, Nanjing 210044, China; 20211249200@nuist.edu.cn (B.Z.); jqding@nuist.edu.cn (J.D.); 2 State Key Laboratory of Millimeter Waves, Southeast University, Nanjing 210018, China; 3 National Key Laboratory of Science and Technology on Space Microwave, China Academy of Space Technology, Xi’an 710100, China; lisheng_cast@163.com

**Keywords:** computerized numerical control (CNC), W-band, multi-polarization, phase compensation, orthogonal mode coupler, square-horn antenna

## Abstract

A wideband multi-polarized square-horn antenna based on an orthogonal mode transducer (OMT) is developed for working in the whole W-band in this paper. The designed antenna is capable of radiating multiple polarization modes as horizontal polarization (HP) and vertical polarization (VP) when as single-port excitation and left-handed circular polarization (LHCP) and right-handed circular polarization (RHCP) when as dual-port excitation, owing to the characteristic of the OMT with the transmitting of orthogonally polarized waves. A CNC-layered fabrication approach is proposed, which means that the antenna prototype integrating with a Boifot-type OMT, turning waveguide, twisting waveguide and phase shifter is divided into three layers along the vertical direction to be fabricated based on computerized numerical control (CNC) technology. In the design, the turning waveguide and twisting waveguide are employed to achieve plane consistency of the antenna branch ports. Furthermore, a phase shifter is designed to compensate the orthogonally polarized waves, which can keep the phase of the orthogonally polarized waves consistent in a wideband frequency range from 75 GHz to 110 GHz. A prototype is fabricated and measured to verify the performance of the proposed multi-polarization antenna, and the measured results agree well with the simulation ones. In the whole W-band, the value of return loss is better than 10 dB of all polarization modes, and the value of AR of the LHCP and RHCP is below 3.5 dB. The maximum gain of the antenna reaches up to 18.8 dBi at 110 GHz. In addition, regarding the layered structure, the possible layered assembly error analysis is discussed, which verifies the feasibility of the layered machining for this antenna.

## 1. Introduction

The millimeter wave has a range from 30–300 GHz; its wavelength is short, and its atmospheric properties are superior to infrared and light waves. Therefore, it is widely used in meteorological detection, remote sensing, imaging and communication [1,2,3,4]. As a key component of the millimeter wave system, the antenna needs to be developed in the multi-function direction to meet the needs of different systems, so the multi-polarization antenna has been widely used in recent years. It is remarkable that the W-band from 75 to 110 GHz is an important frequency window spectrum. Therefore, the research of the multi-polarization antenna in the W-band [5] is of great significance.

At present, the multi-polarized antennas widely used in millimeter wave bands mostly use the combination of the orthogonal mode transducer (OMT) and horn antenna to achieve dual-polarized radiation [6,7,8,9,10,11,12,13,14]. A side-arm OMT machined using computer numerically controlled (CNC) milling technology was proposed in [14], and the measured antenna bandwidth was only 14.2%. This structure is easy to machine; however, due to the existence of high-order modes, this asymmetric OMT inevitably brings the characteristic of narrow impedance width, sacrificing the advantage of a horn antenna. For symmetric OMTs such as Boifot [6] and turnstile types, they have two symmetric planes that can suppress higher-order modes well, so it is easy to obtain broadband characteristics. In the literature [12], there was designed a dual-polarized horn antenna using a turnstile OMT that can work in the whole Ka-band. However, the complex structure dictates that it must be divided into multiple pieces for CNC fabrication. Inevitably, there is an alignment bias problem when assembling between each part, which will affect its performance. Compared with the block method along the central axis of the OMT in [15], the layered machining of the OMT proposed in [16] solves the alignment problem of multiple dimensions, which will further reduce the impact of assembly errors. In addition, other micro-technologies [17,18], such as 3D printing [7] and SU-8 [17], have been successfully used for fabricating dual-polarized antennas or complex OMT structures. Three-dimensional printing [19,20,21] can manufacture extremely complex structures from monomers, which is advantageous for symmetric OMT, but the rough precision is not suitable for millimeter wave antenna processing. At the same time, these precision manufacturing technologies are accompanied by high manufacturing costs. Actually, CNC milling is more common in the millimeter wave band. With the above considerations, this paper adopts Boifot-type OMT to obtain a wideband multi-polarization antenna and fabricate it in layers with CNC.

On the other hand, most of these dual-polarized antennas based on the OMT have low integration due to the limitations of CNC machining; the antenna and the OMT are manufactured separately and then joined together by flanges. In addition, the two ports of these antennas are not on the same plane due to the special structure of the OMT, so the antennas can only work in single-port modes, which is a kind of waste for the other port, which leads to these antennas only achieving two orthogonally linear polarizations (LPs). In the literature, Refs. [10,11] realized the circularly polarized antenna based on the OMT by integrating other passive devices, such as a power splitter and phase shifter; however, their bandwidths were below 10%, and there were no reasonable machining schemes given.

Therefore, in this paper, a wideband multi-polarized horn antenna over the entire W-band is developed. The antenna based on Boifot-type OMT is fabricated in layers using CNC machining technology. In this way, the effect of assembly errors in only one dimension needs to be considered. An E-plane turning waveguide and a 90° twisting waveguide can be integrated into the OMT branch port with CNC layered machining. This structure allows the antenna to work at both single-port and dual-port. During the design process, the phases of the two polarized waves produced by OMT are further studied, and the phase compensation is carried out with an integrated phase shifter. The multi-polarized horn antenna has two feed ports; when a single port of the antenna is fed, the antenna will radiate linear polarization mode (H, V), and when two ports are fed by a 3 dB coupler at the same time, the antenna will radiate circular polarization mode (LHCP, RHCP). In addition, the effects on the antenna due to the assembly offset in CNC layer machining were studied to verify the feasibility of layer machining.

## 2. Antenna Description

**Figure 1:**
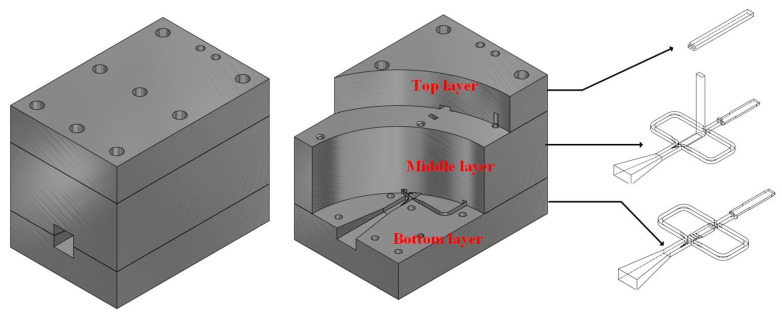
Configuration of the designed multi-polarization horn antenna based on Boifot-type OMT.

Figure 1 illustrates the designed multi-polarization horn antenna based on Boifot-type OMT, which is realized by superimposing three aluminum block layers. The bottom layer and bottom layer consist of a horn antenna integrated with Boifot-type OMT, turning waveguide, twisting waveguide and phase shifter, and the middle layer has a through-waveguide for connecting the top layer; the top layer is an E-plane turning waveguide. Computer numerical control (CNC) milling technology is employed to process the designed antenna metal mechanical processing, which lowers the final cost.

**Figure 2:**
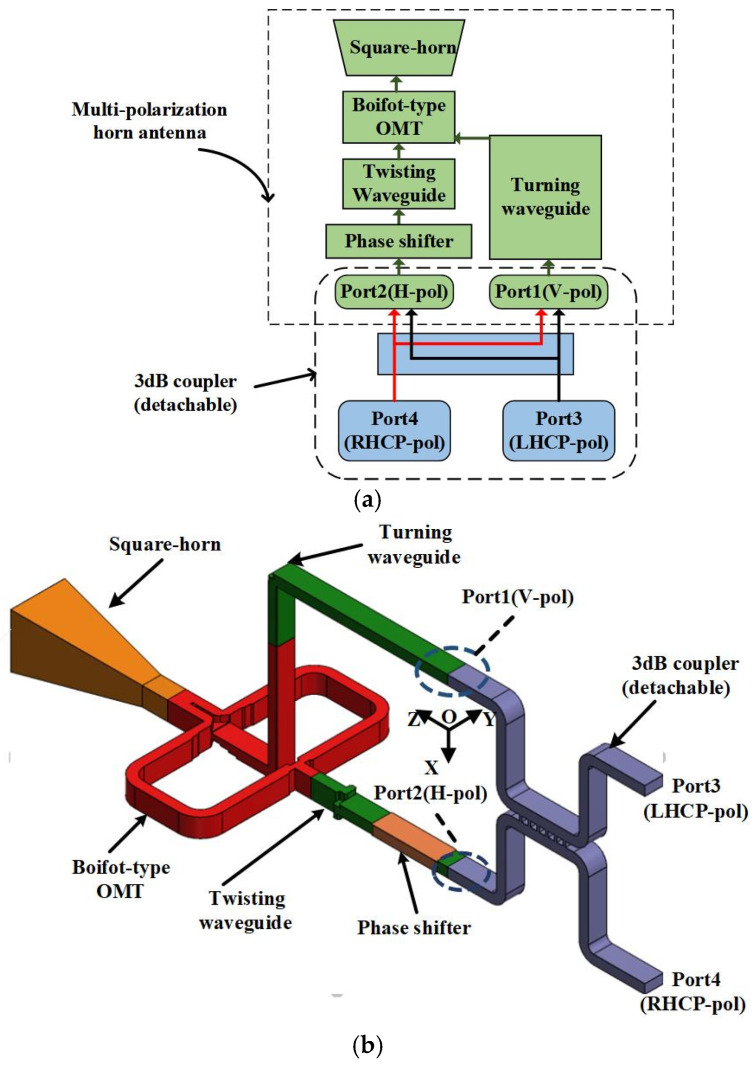
(**a**) Specific principal block diagram of the proposed multi-polarization horn antenna with a detachable 3 dB coupler (the directions of the arrows can be reversible). (**b**) Cavity structure of the antenna with a detachable 3 dB coupler.

The specific principal block diagram of the multi-polarization horn antenna is shown in Figure 2a. Due to its broadband and simple structural characteristics, the square horn is used as the radiation part. It is integrated with the Boifot-type OMT, which can transmit orthogonally polarized waves. Different from other multi-polarization horn antennas based on OMT, two branch ports of the OMT in this design have planar consistency by introducing a turning waveguide and twisting waveguide, which makes the antenna able to be integrated and work in dual port mode. A phase shifter is used to compensate for the orthogonally polarized waves generated by an OMT so that the orthogonally polarized waves will keep their phase coincidence. When only Port1 (Port2) is fed, the antenna will transmit the VP (HP) mode. When Port1 and Port2 are fed with a (±) 90° phase difference at the same time, the antennas will transmit the LHCP (RHCP) mode. Therefore, a detachable 3 dB coupler is needed as a power divider, and the coupler is connected to the antenna when the antenna needs to work under circularly polarized radiation, and the coupler is not needed when the line polarized radiation is required. The cavity structure of the antenna is presented in Figure 2b; Port1 and Port2 are in the XOY plane, and there is a detachable 3 dB coupler connected with it. The antenna consists of four parts: (1) Boifot-type OMT; (2) twisting waveguide and turning waveguide; (3) phase shifter; (4) square-horn antenna. And a detachable 3 dB couple is designed for feeding the antenna.

## 3. Antenna Design

### 3.1. Boifot-Type OMT

**Figure 3:**
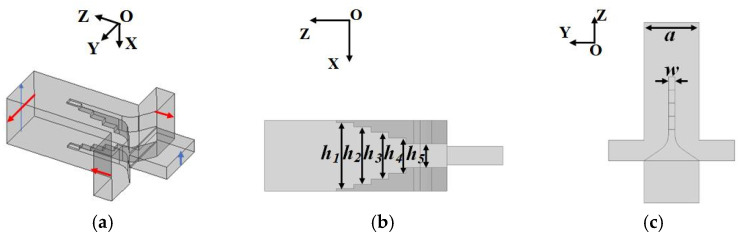
Geometries of the Boifot junction. (**a**) Perspective view. (**b**) Side view in XOZ-plane. (**c**) Top view in YOZ-plane.

The OMT is the indispensable component in this multi-polarization horn antenna. The performance of the Boifot-type OMT is mainly determined by the Boifot junction shown in Figure 3a. The operating principle is that the TE_10_ and TE_01_ modes can be synthesized/separated in the common Port4. The vertical TE_10_ mode transfers between Port1 and Port4 and is concentrated at the center of a double ridge. The horizontal TE_01_ mode is divided into two side ports. Because of the symmetry, higher-order modes are suppressed, and the transmission performance and isolation are excellent in broadband. The stepped double ridge is optimized for a low reflection coefficient. A detailed geometric parameter of the double-ridge and common port is indicated in Figure 3b,c and Table 1. The specific size of the OMT design is less than the machining accuracy of CNC machine tools, and due to the existence of the double ridges, the structure needed to be split into two blocks for machining to accommodate CNC milling technology. This split-block machining method gives the OMT the possibility of integrating with a double-corner-cut twisting waveguide.

**Figure 4:**
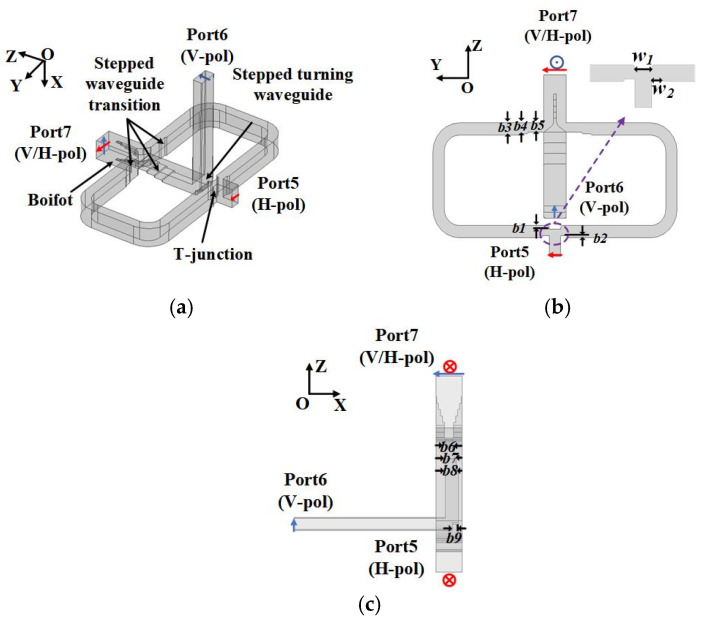
Geometries of the Boifot-type OMT. (**a**) Perspective view. (**b**) Side view in YOZ-plane. (**c**) Side view in XOZ-plane.

Figure 4 shows the designed Boifot-type OMT attached with a detailed geometric parameter in Table 2. The narrow edges of the branch ports of the Boifot junction are decreased to improve the isolation, so a stepped waveguide is needed for transition. The E-plane T-junction and stepped turning waveguide discussed in Part B are designed as a branch channel, which only transforms the basic mode TE_10_.

**Figure 5:**
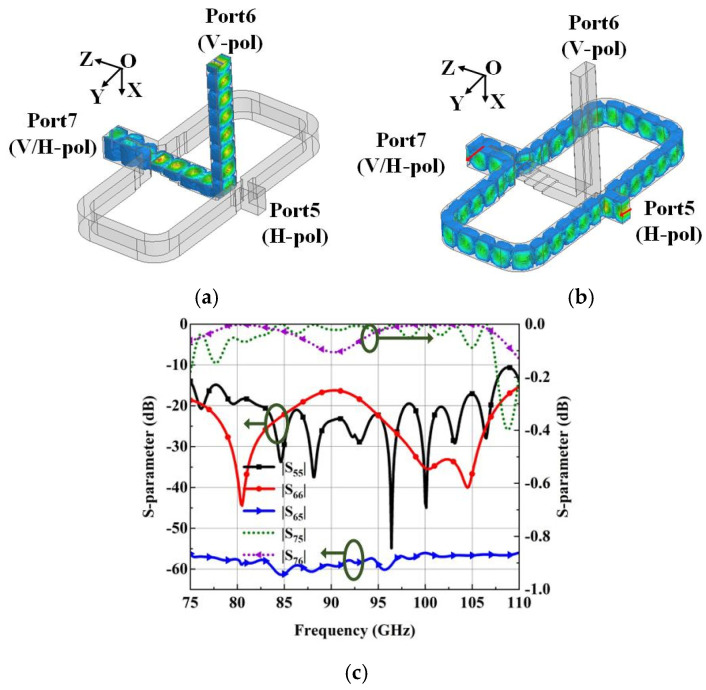
Simulated results of the OMT. (**a**) Electric field distributions at 92.5 GHz with Port6 as excitation. (**b**) Electric field distributions at 92.5 GHz with Port5 as excitation. (**c**) S-parameter of the OMT.

The simulated electric field distributions of the OMT with different excitation from Port5 or Port6 at 92.5 GHz are plotted in Figure 5a,b. What can be seen is the waves from branch Port5 and Port6 all transfer in basic mode to common Port7 with high isolation, which confirms the operating principle of the OMT. Furthermore, a simulated reflection coefficient of less than −10 dB can cover the whole W-band (from 75 to 110 GHz, bandwidth of 37.8%), as is shown in Figure 5c. The transfer loss is less than 0.4 dB, and the isolation is above 56 dB in the whole operating band.

### 3.2. Twisting Waveguide and Turning Waveguide

**Figure 6:**
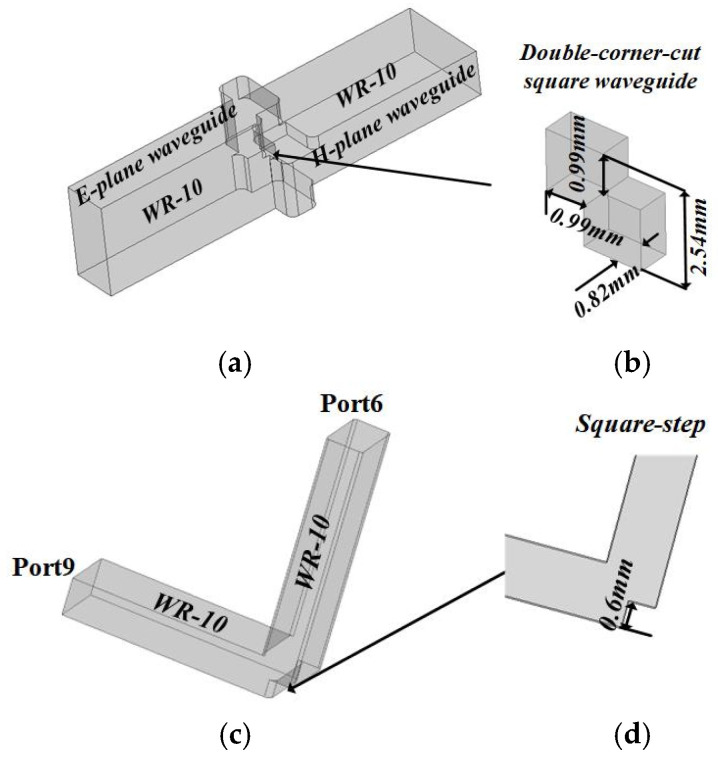
Geometries of the twisting waveguide and turning waveguide (key parameters are provided). (**a**) Perspective view of twisting waveguide. (**b**) Twisting section. (**c**) Perspective view of turning waveguide. (**d**) Turning section.

As with the OMT already reported [6,7,8], the branch ports of the Boifot OMT design above are also not in the same plane. Due to the limitation of machining methods, the consistency of the OMT branch port plane is almost not realized in the previous work, causing the antenna based on the OMT to be reported as only achieving single-port operation mode. In order to overcome this difficulty, in this paper, the OMT can integrate with a twisting waveguide and turning waveguide using layered CNC machining so that the two-port planar consistency is realized, and the antenna can work in the two-port mode. Port5 is in the YOX-plane, and Port6 is in the YOZ-plane, as shown in Figure 4; to enable Port6 and Port5 to be in the same YOZ-plane, an E-plane step rotation waveguide is designed. The E-plane turning waveguide adopts a step structure to adapt the CNC H-plane milling machining. Furthermore, there is another problem in that the electric field direction of the two branch ports has a 90° difference, so a twisting waveguide is needed. The single double-corner-cut twisting waveguide is proposed in [22], which can be easily machined by means of a split block based on the current CNC-milling technology. Considering that the Boifot-type OMT is also processed through segmentation blocks, this twisting waveguide can be well integrated with the OMT on a block to process. Through this integration, the electric field of Port5 can rotate 90°. The detailed configurations are shown in Figure 6.

**Figure 7:**
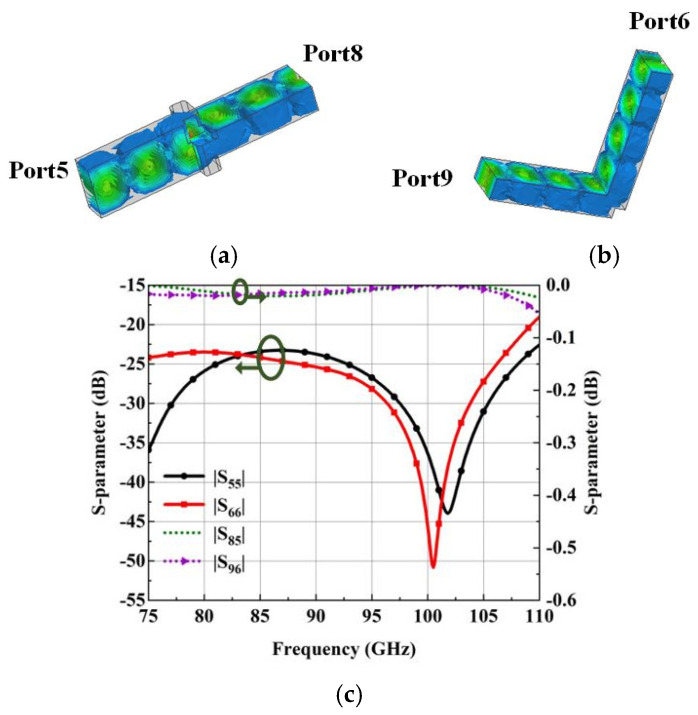
Simulated results of the twisting waveguide and turning waveguide. (**a**) Electric field distributions at 92.5 GHz with Port8 as excitation. (**b**) Electric field distributions at 92.5 GHz with Port9 as excitation. (**c**) S-parameter.

The simulated electric field distributions of the twisting waveguide at 92.5 GHz are plotted in Figure 7a. The double-corner-cut square waveguide can first rotate vertical polarization in the H-plane waveguide by 45°, then convert the 45° polarized wave to the horizontal polarization in the E-plane waveguide. Figure 7b is the simulated electric field distribution of the turning waveguide at 92.5 GHz. Through the square step, the TE_10_ mode wave can achieve better matching transmission in the turning waveguide. Figure 7c is the simulated transmission characteristic of the twisting waveguide and turning waveguide. In the whole W-band, the reflection coefficient is less than −20 dB, and the transfer loss is less than 0.1 dB in the whole W-band.

### 3.3. Phase Shifter

**Figure 8:**
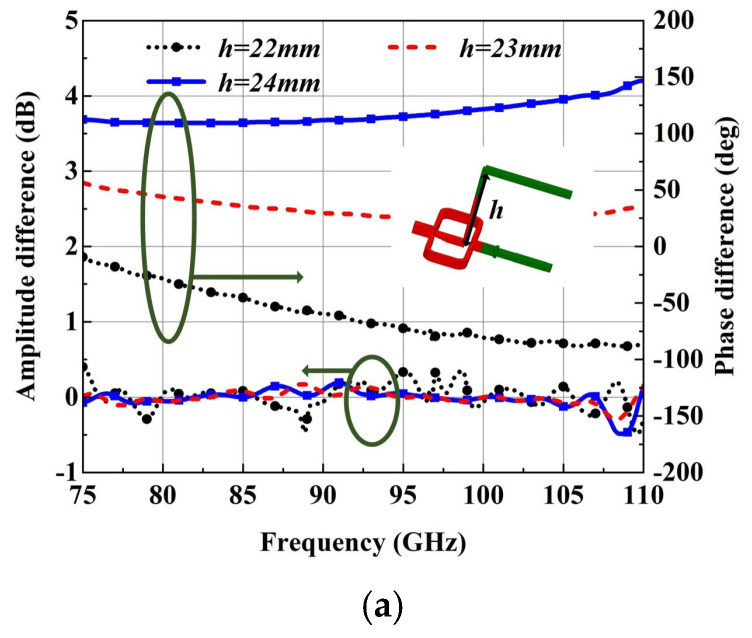

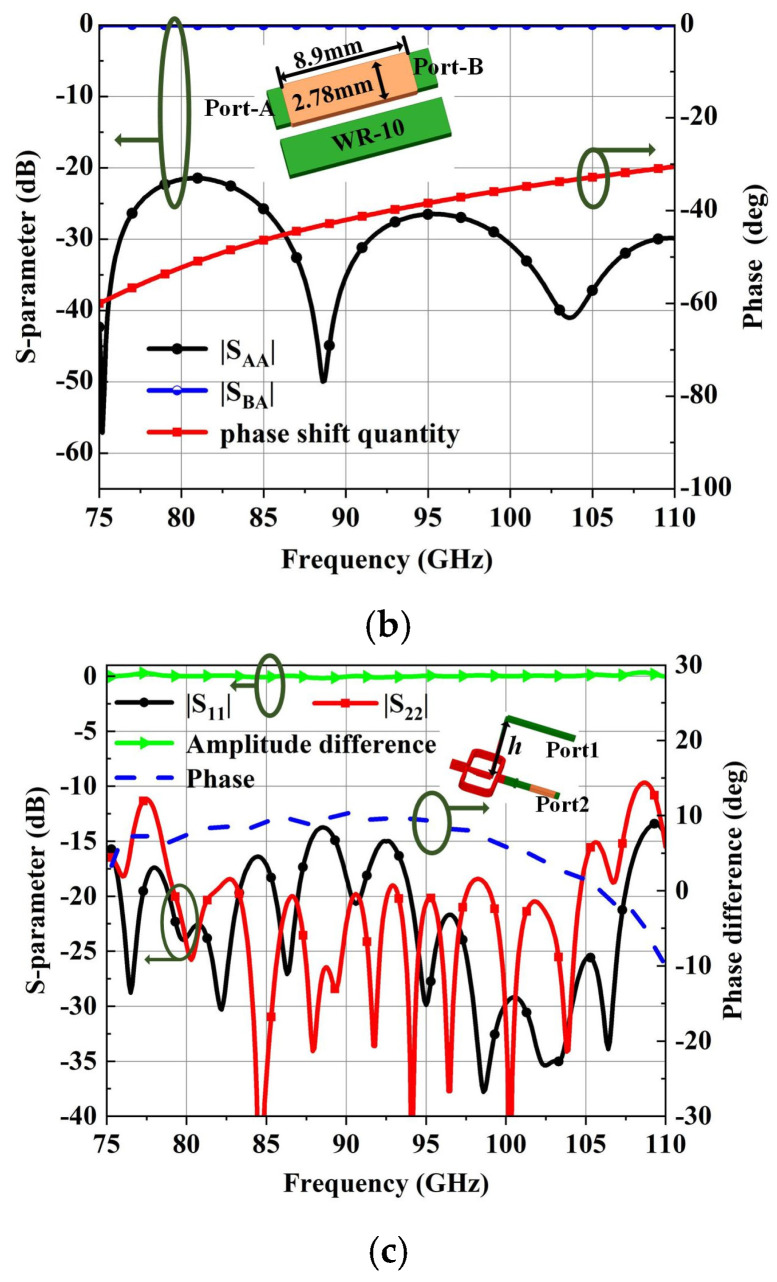
(**a**) Parametric study of the OMT with port plane consistency (no phase shifter). (**b**) Simulated S-parameter and phase shift quantity of the phase shifter. (**c**) Simulated results of the proposed OMT with phase shifter.

In addition to solving the problem of port plane consistency, how to realize wideband circular polarization in dual-port mode is another focus of this paper. In order to achieve broadband circular polarization, the quadrature polarized wave must first maintain a relatively stable phase difference of 0° in the whole W-band. Generally, in the OMT section, the orthogonally polarized waves produced by the OMT will have phase differences. However, the phase difference is not stable in broadband due to the different transmission paths of HP and VP waves. Parameter h, connected with a transmission path in this study in W-band, is shown in Figure 8a, which reveals the effects of the length of the VP transmission path on phase and amplitude differences. It can be seen that parameter h has a great effect on the phase difference of the orthogonally polarized wave transfer in the OMT with port plane consistency. The amplitude difference is almost unaffected and stable at (0 ± 0.5) dB, while the phase difference of the quadrature polarization wave varies greatly when h varies. Relatively steady phase difference (25–56°) performance is obtained in W-band for an h of around 23 mm. To keep the phase of the orthogonally polarized wave coinciding, a phase shifter is needed to compensate for the phase difference.

The phase velocity of the TE10 mode is determined by the wide-edge and narrow-edge dimensions of the rectangular waveguide. A phase shifter is designed by expanding the wide edge of the WR-10, which can change the phase velocity of the TE10 mode, and it can be easily integrated into the branch path. The standard rectangular waveguide WR-10 with the same transmission length as the phase shifter that is designed to compare with the phase shifter is shown in Figure 8b. It can be seen that the phase shift quantity is 40° at 92.5 GHz, and it varies with a steady trend (33–55°), which is matched with the orthogonally polarized wave phase difference in the whole W-band. Therefore, using this kind of phase shifter to adjust the phase difference of the orthogonally polarized waves can keep it at about 0° in the whole W-band. After optimization of parameter h, the simulated results are shown in Figure 8c, and it can be seen that the reflection coefficient of Port1 and Port2 is less than −10 dB in the whole W-band, and the amplitude difference of the orthogonally polarized waves is at (0 ± 0.3) dB. This suggests that the phase shifter will not damage the transmission performance of the OMT. The phase difference of the orthogonally polarized waves is at (0 ± 10°) in the whole frequency band. Based on this work, the OMT can nearly transfer the orthogonally polarized waves with constant amplitude and phase in the whole W-band.

### 3.4. Square-Horn Antenna

**Figure 9:**
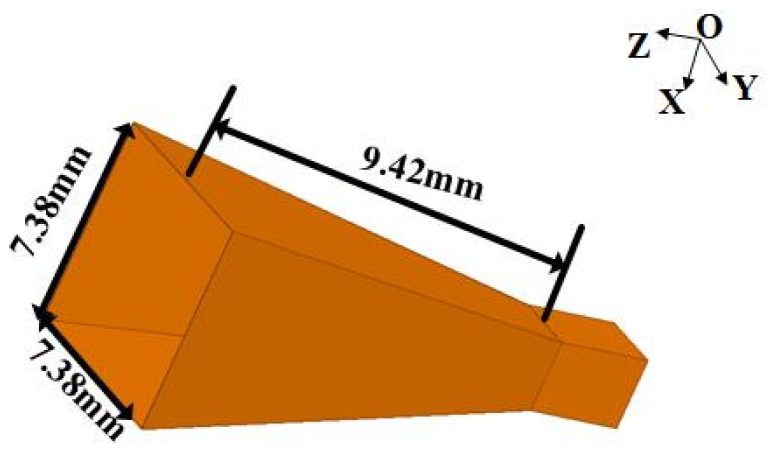
Geometries of the square-horn antenna.

Another key component in this multi-polarization system is the radiating antenna, which can be integrated with the square waveguide. Therefore, a horn antenna is suitable for this system because it can be seen as a waveguide with an open aperture, and it still transmits TE_10_ (V-pol) and TE_01_ (H-pol) modes, which are the basic modes of square waveguides internally, and its radiation characteristic is mainly decided by the size of the aperture. The aperture size and axial length of the horn are the basic parameters to determine the gain of the horn antenna. The length of the horn is also crucial to the impedance matching of the antenna. A suitable aperture size and axial length of the horn are calculated and optimized for full W-band operation with a minimum gain of 15 dB. Considering the transmission of the orthogonally polarized waves in the horn component and the radiation performance, a horn with a square aperture (7.38 mm × 7.38 mm) is finalized, which is beneficial to keep the same amplitude and phase difference of the orthogonally polarized waves (V-pol and H-pol). The geometries of the square horn are shown in Figure 9.

### 3.5. 3 dB Coupler

**Figure 10:**
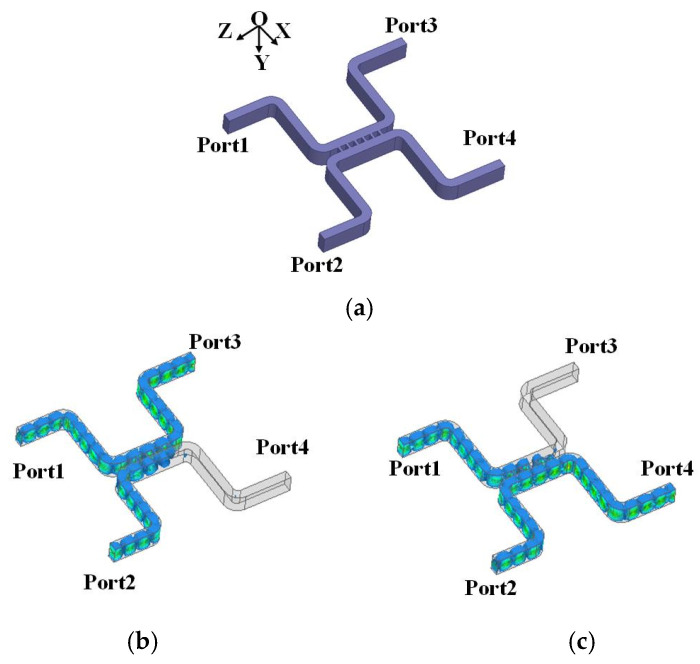
(**a**) Geometries of the 3 dB coupler. (**b**) A total of 92.5 GHz electric field distributions when Port3 is fed. (**c**) A total of 92.5 GHz electric field distributions when Port4 is fed.

To achieve circularly polarized radiation, a 3 dB coupler is employed to realize a power divider with a 90° phase difference. This is different from the traditional 3 dB branch waveguide coupler proposed in [23], which uses a different gap. A 3 dB coupler with a unified gap that can be manufactured easily is adopted in this paper. When the branch is added, the coupler will feature a low amplitude imbalance [24]; however, with the number of branches increasing, the gap width will become smaller. Considering the processing difficulty in W-band, a 6-branch coupler is designed. The geometries of the 3 dB coupler are shown in Figure 10a, and electric field distributions at 92.5 GHz are shown in Figure 10b,c.

When only Port3 (Port4) is fed, the signal will be equally distributed to Port1 and Port2 with a ±90° phase difference [see Figure 10b,c]; then the V-pol and H-pol will have a ±90° phase difference and almost the same amplitude. At this time, the antenna will spread LHCP (RHCP) waves.

**Figure 11:**
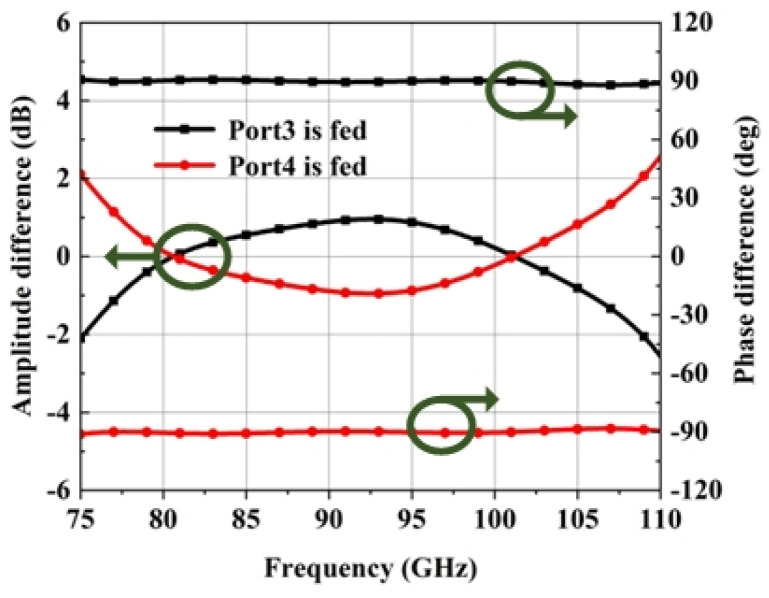
Simulated amplitude and phase difference of the 3 dB coupler output ports (Port1 and Port2).

The simulated results of the coupler are shown in Figure 11. It can be seen that Port1 and Port2 will obtain signals with almost the same amplitude fluctuation within 2 dB and a ±90° phase difference in the whole W-band. Therefore, the antenna can realize LHCP or RHCP radiation in the whole W-band when fed using this 3 dB coupler.

## 4. Fabrication Discussion and Measurement

**Figure 12:**
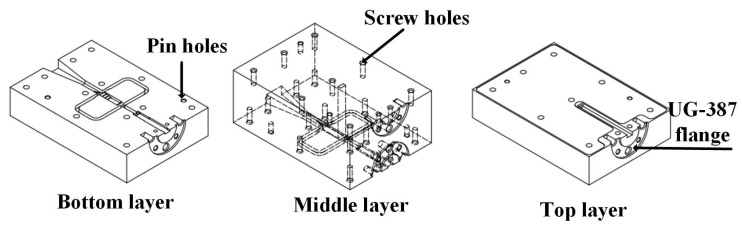
Layered multi-polarization horn antenna structure and the machining blocks.

**Figure 13:**
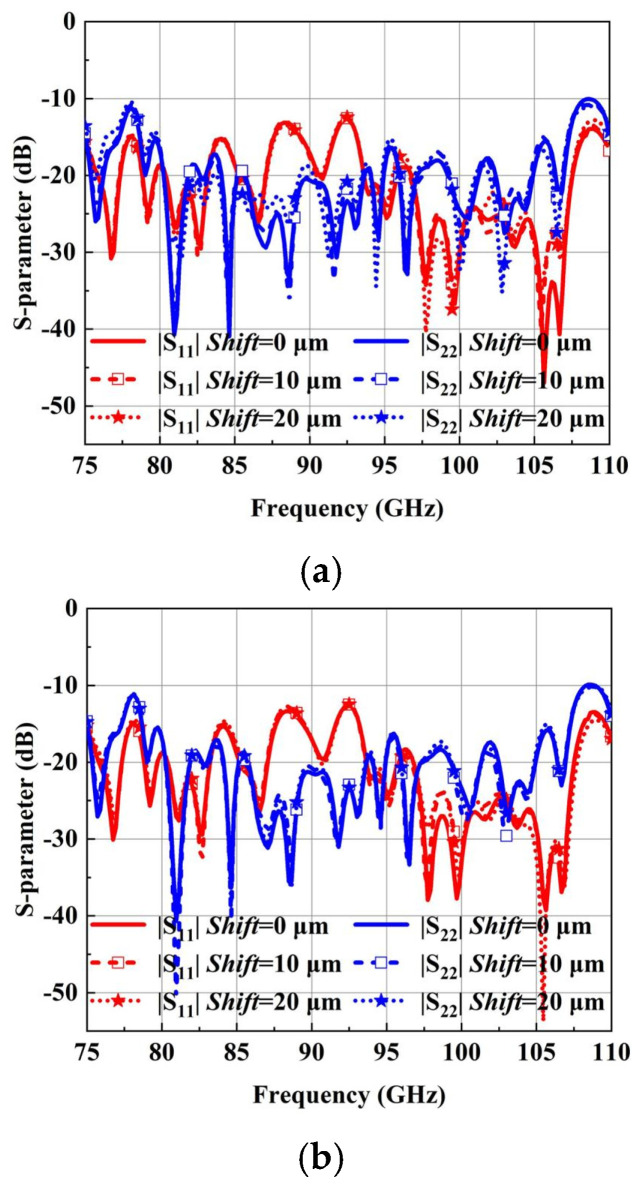
The antenna return loss. (**a**) Effects of bottom layer shifting errors. (**b**) Effects of top layer shifting errors.

**Figure 14:**
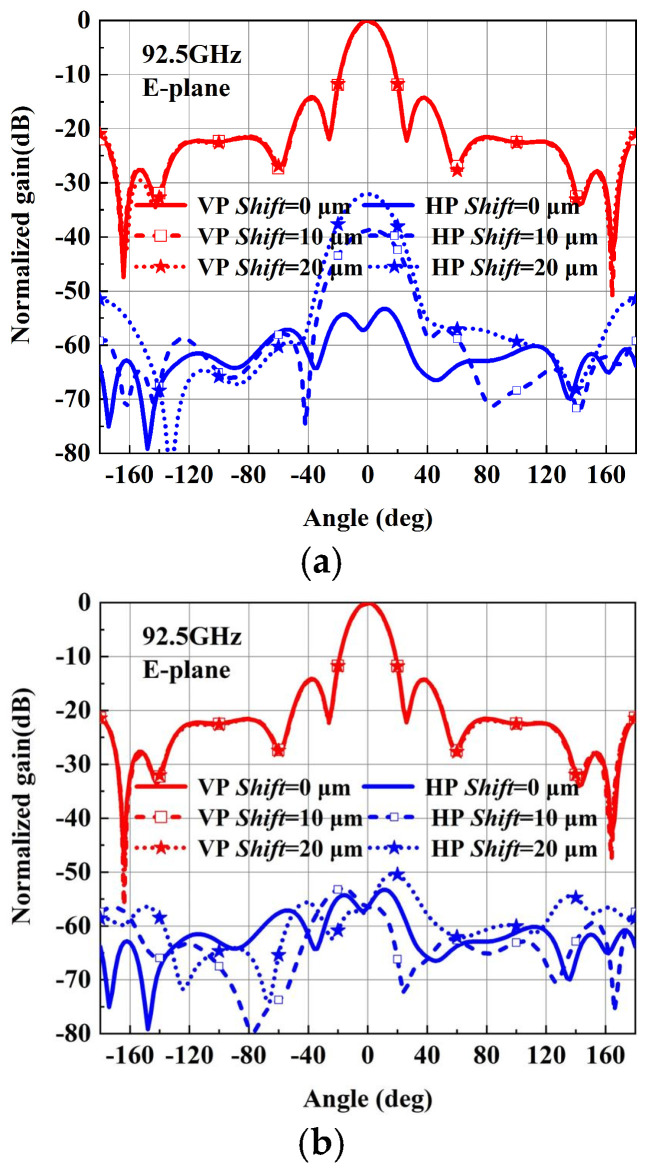

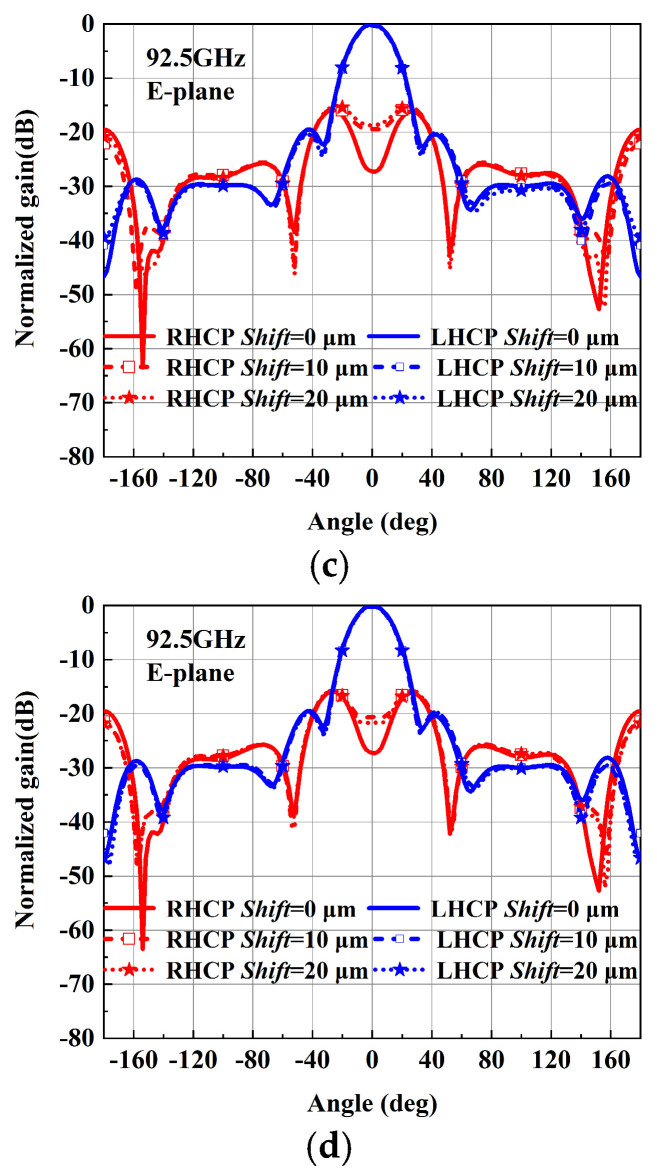
The antenna radiation performance. (**a**) Effects of bottom layer shifting errors on VP. (**b**) Effects of top layer shifting errors on VP. (**c**) Effects of bottom layer shifting errors on LHCP (ideal feed). (**d**) Effects of top layer shifting errors on LHCP (ideal feed).

Based on the above design, the antenna integrating with Boifot-type OMT, turning waveguide, twisting waveguide and phase shifter are fabricated in a layered aluminum block using a CNC milling technique. According to the structure of the antenna, it can be vertically divided into 3-layer configurations, as shown in Figure 12; the three layers are aligned with pins and joined with screws. All the WR-10 ports are configured with a standard UG-387 flange to realize the connection with the 3 dB coupler. Considering the offsetting errors between layers has become a crucial problem in this layered structure. The simulation of layer shifting on the 3-layer antenna performance is carried out, as shown in Figure 13. A shift may exist between every block layer due to the split-block assembling; the effects of the bottom and top block shifts are simulated, respectively. However, whether the layer is moved 10 μm or 20 μm, the return loss of the antenna in the whole W-band is less than 10 dB, which indicates that the working bandwidth of the antenna is not affected by the assembly errors within 20 μm. The radiation performance is shown in Figure 14; it can be seen that the main polarization pattern of the antenna is not affected by assembly deviation, regardless of the linear polarization radiation mode or the circular polarization radiation mode. But, as the assembly deviation increases, the cross-polarization deteriorates, and the linear polarization radiation pattern shows up more clearly. However, the impact is still acceptable when the shift changes by 20 μm. Fortunately, the aligned accuracy of the pins in the current CNC-machining is less than 20 μm, which makes it clear that the approach of using CNC to fabricate complex antennas in layers proposed in this paper is feasible. It can be found by these simulations that the offset of the bottom layer has a more severe effect on the antenna radiation performance, which is worth paying more attention to when assembling.

**Figure 15:**
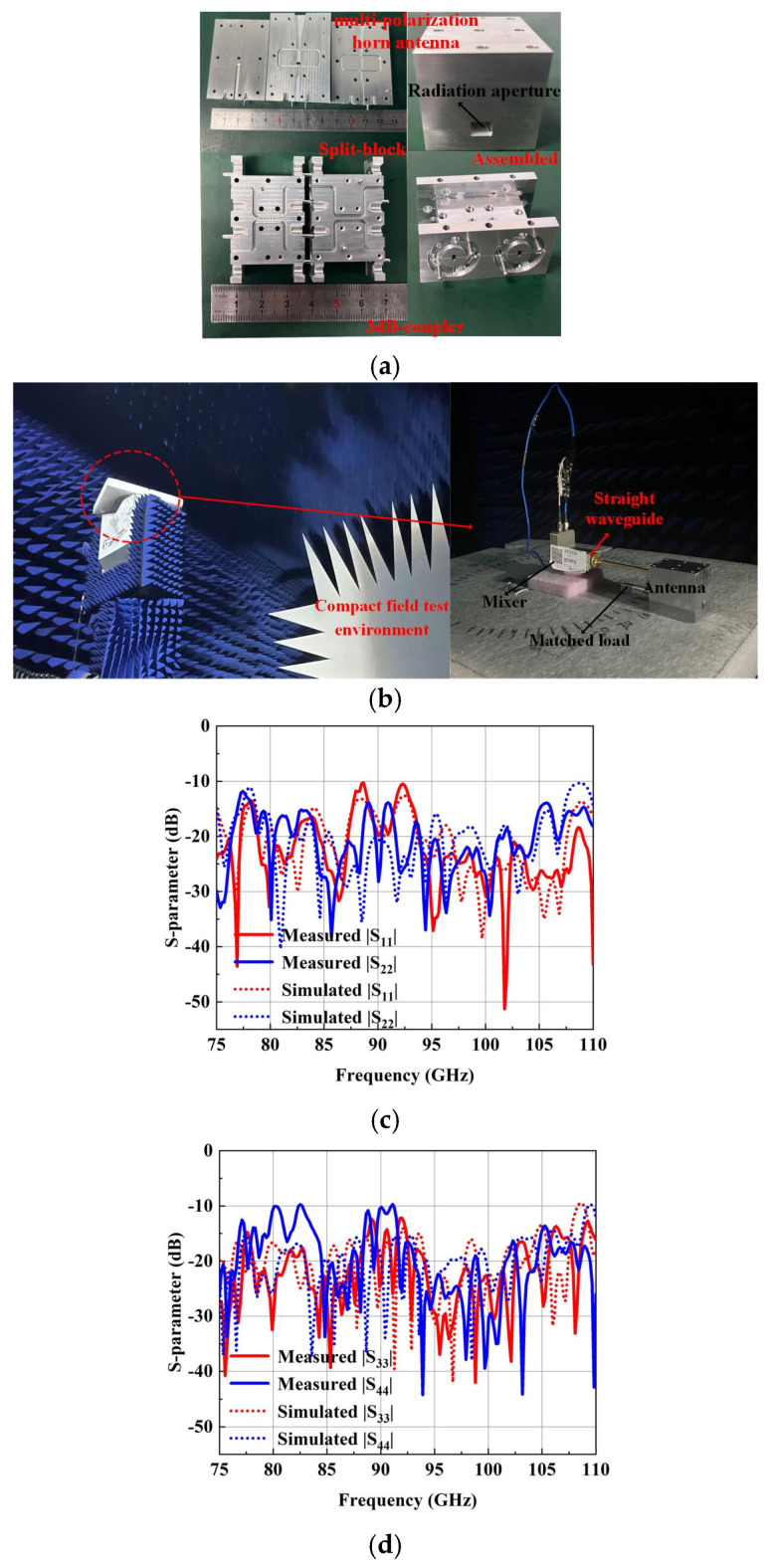
(**a**) Photograph of the proposed multi-polarized horn antenna and 3 dB coupler. (**b**) The compact field test environment. (**c**) Measured and simulated reflection coefficient of the V-pol and H-pol modes. (**d**) Measured and simulated reflection coefficient of the LHCP and RHCP modes.

The S-parameter is measured using the Agilent N5260A Vector Network Analyzer (VNA) with an OML W-band extension module. The measured results compared with simulated results are shown in Figure 15c,d. It is found that the measured results agree well with the simulation results. The reflection coefficients of the four polarization modes (Port1 to Port4) are all better than 10 dB in the whole W-band, with a relative bandwidth of 37.8%.

**Figure 16:**
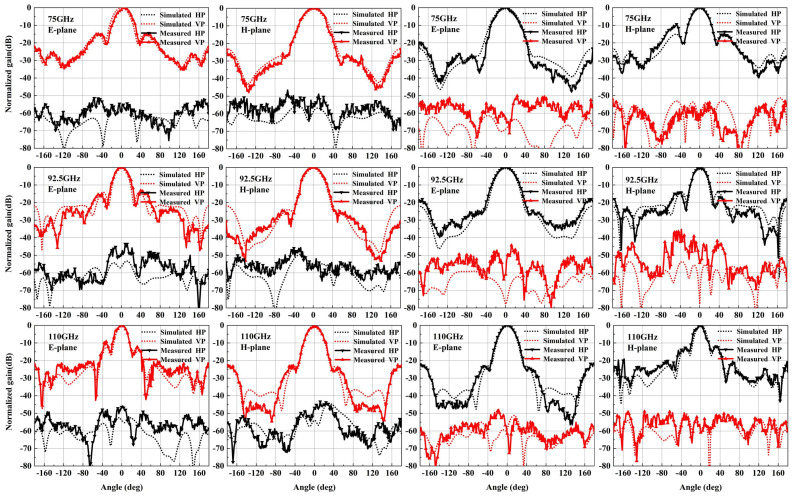
Measured and simulated radiation patterns for linear polarization modes. (The left six figures show the gain pattern for VP mode, while the right figures represent the gain pattern for HP).

**Figure 17:**
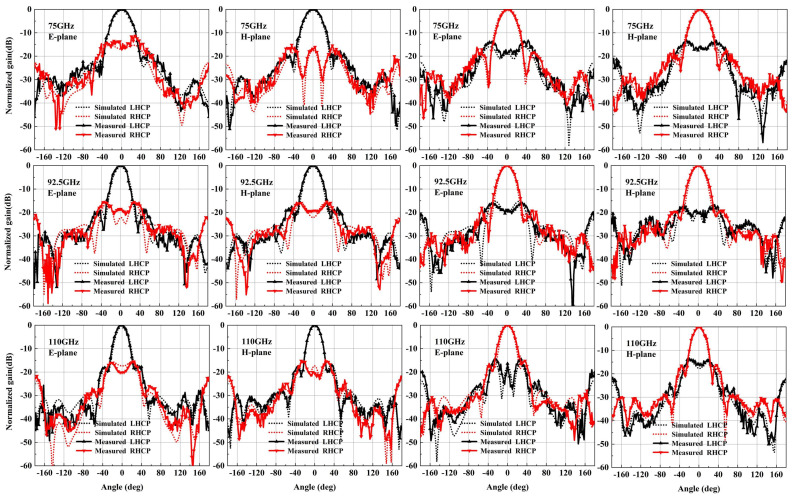
Measured and simulated radiation patterns for circular polarization modes. (The left six figures show the gain pattern for LHCP mode, while the right figures represent the gain pattern for RHCP).

**Figure 18:**
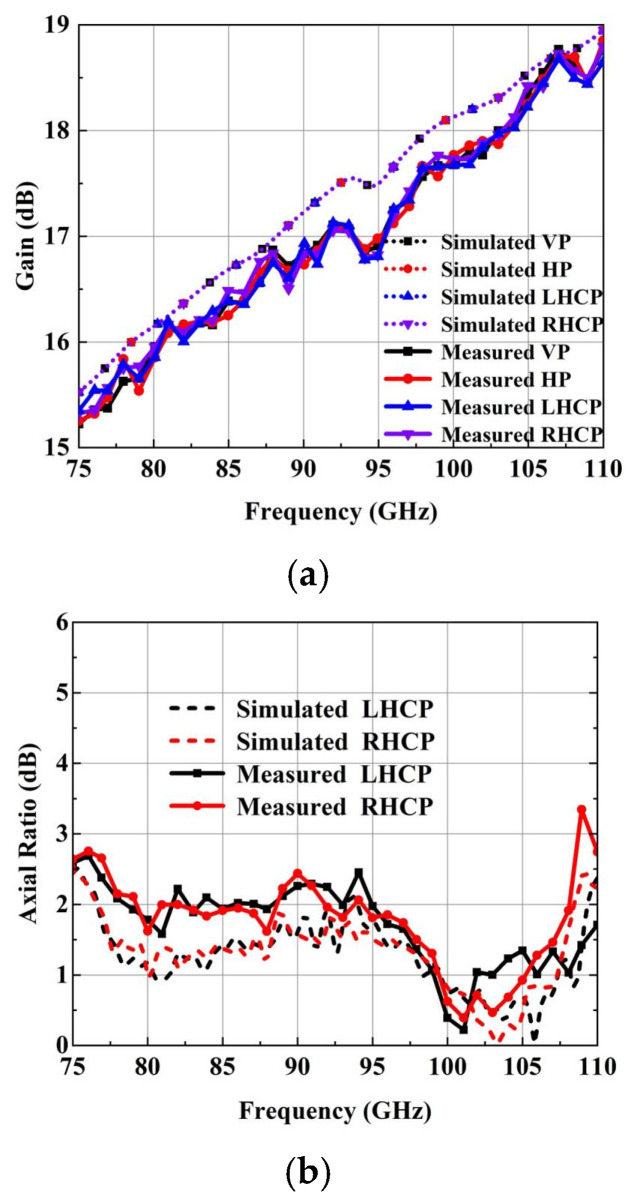
Measured and simulated radiation performance of the designed antenna. (**a**) The peak gain of the antenna. (**b**) The AR of the LHCP and RHCP.

Afterward, a compact field test environment, shown in Figure 15b, is used to capture the radiation performance of the designed horn. As shown, one input port of the designed antenna is connected to the mixer through a WR-10 straight waveguide, and another port is connected to the matched load to absorb the interference signal. The signal received by the designed antenna is transmitted to the RF receiver through the mixer. All of the signal is produced by a W-band multiplier and radiates through the standard gain horn antenna. The measured normalization radiation patterns of the four polarization modes are in good agreement with the simulated results. It is seen in Figure 16 and Figure 17 that the cross-polarization for VP and HP is less than −43 dB at 75 GHz, 92.5 GHz and 110 GHz. Meanwhile, the cross-polarization for LHCP and RHCP is less than −17 dB at 75 GHz, 92.5 GHz and 110 GHz. The gain of the four polarization modes is basically coincidental due to the use of the same radiation aperture, as shown in Figure 18a; the maximum gain is 18.8 dBi, which is slightly lower than the simulated results. It is also observed in Figure 18b that the AR for LHCP is less than 3 dB in the whole W-band, and for RHCP, it is less than 3.5 dB. The measured AR is slightly higher than the simulated AR of the two circular polarization modes, possibly due to the measuring and machining errors.

Table 3 shows a comparison of this antenna with some reported multi-polarization antennas based on the OMT. Obviously, compared with these antennas, the proposed antenna has considerable advantages in radiation polarization modes, which can adapt to more applied scenarios. Meanwhile, it maintains a wide bandwidth and steady radiation performance, which works in the whole W-band.

## 5. Conclusions

In this paper, a wideband dual-port multi-polarization horn antenna with layered machining is designed by using CNC milling technology. It can work at VP, HP, LHCP and RHCP modes for different port operations. The antenna is fabricated and measured, and the results show that an antenna impedance bandwidth of 37.8% can cover the whole W-band; the cross-polarization of VP and HP is less than −43 dB. The AR of the LHCP and RHCP is less than 3.5 dB from 75 GHz to 110 GHz, which shows that the antenna has stable circularly polarized radiation. The main contribution of this work is to propose that an antenna radiates VP, HP, LHCP and RHCP modes with a wide working bandwidth and stable radiation performance, which can fulfill the requirement of a multi-polarization radiometer for atmospheric sounding. In addition, the feasibility of the layered integration of this CNC machined antenna has been verified, which can promote the application of CNC milling technology in the layered machining of complex structures.

## Figures and Tables

**Table 1:** Dimensions of the Boifot junction (Unit: mm).

*a*	*w*	*h*_1_	*h*_2_	*h*_3_	*h*_4_	*h*_5_
2.54	0.3	2.38	2.08	1.68	1.26	0.86

**Table 2:** Dimensions of the Boifot-type OMT (Unit: mm).

*w*_1_	*w*_2_	*b*_1_	*b*_2_	*b*_3_	*b*_4_	*b*_5_	*b*_6_	*b*_7_	*b*_8_	*b*_9_
1.3	0.8	0.4	0.2	1.27	1.13	0.97	0.68	0.86	1.16	0.6

**Table 3:** Comparison with some reported muti-polarization antenna.

Ref	fc	Bandwidth	Pol Modes	Gain	Tech
[6]	78.5 GHz	19%	VP, HP	—	CNC
[9]	85 GHz	4.7%	VP, HP	14 dBi	PCB
[25]	95 GHz	31.5%	VP, HP	11.1 dBi	3D-printing
This work	92.5 GHz	37.8%	VP, HP, LHCP, RHCP	18 dBi	CNC

## Data Availability

Data are available when authors are asked.
